# Aerobic Exercise Modulates Sirt1 to Alleviate Diabetic Arterial Remodeling

**DOI:** 10.3390/ijms27198750

**Published:** 2026-09-30

**Authors:** Yanzhong Liu, Dan Wang, Yiping Liu

**Affiliations:** 1 School of Physical Education and Health, Henan University of Chinese Medicine, Zhengzhou 450046, China; liu_yz2020@126.com; 2 Provincial University Key Laboratory of Sport and Health Science, School of Physical Education and Sport Sciences, Fujian Normal University, Fuzhou 350117, China

**Keywords:** type 2 diabetes mellitus, exercise, Sirt1, vascular smooth muscle cell, metabolic reprogramming

## Abstract

Metabolic reprogramming is a pivotal driver of diabetic vascular remodeling by inducing the phenotypic transition of vascular smooth muscle cells (VSMCs). This study demonstrated that in the aortas of diabetic mice, the medial layer was significantly thickened, with disordered arrangement of collagen and elastic fibers, downregulated expression of the contractile marker α-smooth muscle actin (α-SMA), and upregulated expression of the synthetic marker osteopontin (OPN). These pathological changes were accompanied by suppressed Sirtuin 1 (Sirt1) expression and increased glycolytic enzyme activity. Our results further demonstrated that an 8-week treadmill exercise intervention effectively restored aortic Sirt1 levels in diabetic mice, ameliorated mitochondrial damage, normalized elevated glycolysis in VSMCs, and attenuated their transition to a synthetic phenotype. Collectively, these findings demonstrate that aerobic exercise-induced improvements in diabetic vascular remodeling are accompanied by alterations in Sirt1 expression, glucose metabolism, mitochondrial morphology, and the VSMC phenotype.

## 1. Introduction

Vascular complications represent the primary threat to the quality of life in patients with type 2 diabetes mellitus (T2DM), with approximately 80% of diabetic individuals experiencing varying degrees of macrovascular complications [1]. Structural remodeling is a key contributing factor that ultimately leads to vascular complications [2]. VSMCs are the primary cell type found in blood vascular walls. In the diabetic state, the transformation of VSMCs to a synthetic phenotype promotes cell proliferation, migration, and enhances cell resistance to apoptosis. The disruption of the balance between VSMC proliferation and apoptosis is a key factor in vascular dysfunction and structural remodeling [3]. Alterations observed in bioenergetics are a key feature of the phenotypic transformation of VSMCs, reflected by an increase in the contribution of glycolysis to cellular energy supply, enhanced glucose uptake, and increased activity of glycolytic enzymes, including hexokinase 2 (HK2) and lactate dehydrogenase (LDH). These changes are essential for sustaining rapid cell proliferation and migration. In contrast, mitochondrial oxidative metabolism of pyruvate is inhibited [4]. This shift toward increased glycolysis under aerobic conditions is referred to as metabolic reprogramming [5]. As an end product of glycolytic metabolism, lactate has been shown to be an important signaling molecule involved in mitochondrial homeostasis and metabolic regulation, accelerating the transition of VSMCs to a synthetic phenotype. Emerging evidence indicates that lactate metabolism is mechanistically linked to Sirt1 expression. Sirt1 is a highly conserved, NAD^+^-dependent deacetylase that regulates mitochondrial function, glucose homeostasis, and cellular redox balance—and plays a critical role in vascular endothelial cell function, VSMC proliferation, and other key vascular pathologies [6,7].

Aerobic exercise has been shown to be an effective non-pharmacological strategy for glycemic control and cardiovascular risk reduction in patients with diabetes, and to modulate the expression of glycolytic enzymes such as HK2 and LDH, thereby improving metabolic flexibility in individuals with type 2 diabetes [8]. In other models, exercise inhibits glycolytic metabolism and lactate production by activating Sirt1, thereby restoring mitochondrial function [6]. However, its role and underlying mechanisms in vascular cells remain unclear. Therefore, this study investigates the effects of aerobic exercise on aortic VSMC phenotype, glucose metabolism, and the expression of Sirt1 in diabetic mice, aiming to provide novel insights into the prevention and treatment of diabetic vascular remodeling through aerobic exercise.

## 2. Results

### 2.1. Exercise Improves Glucose Metabolism in T2DM Mice

**Figure 1:**
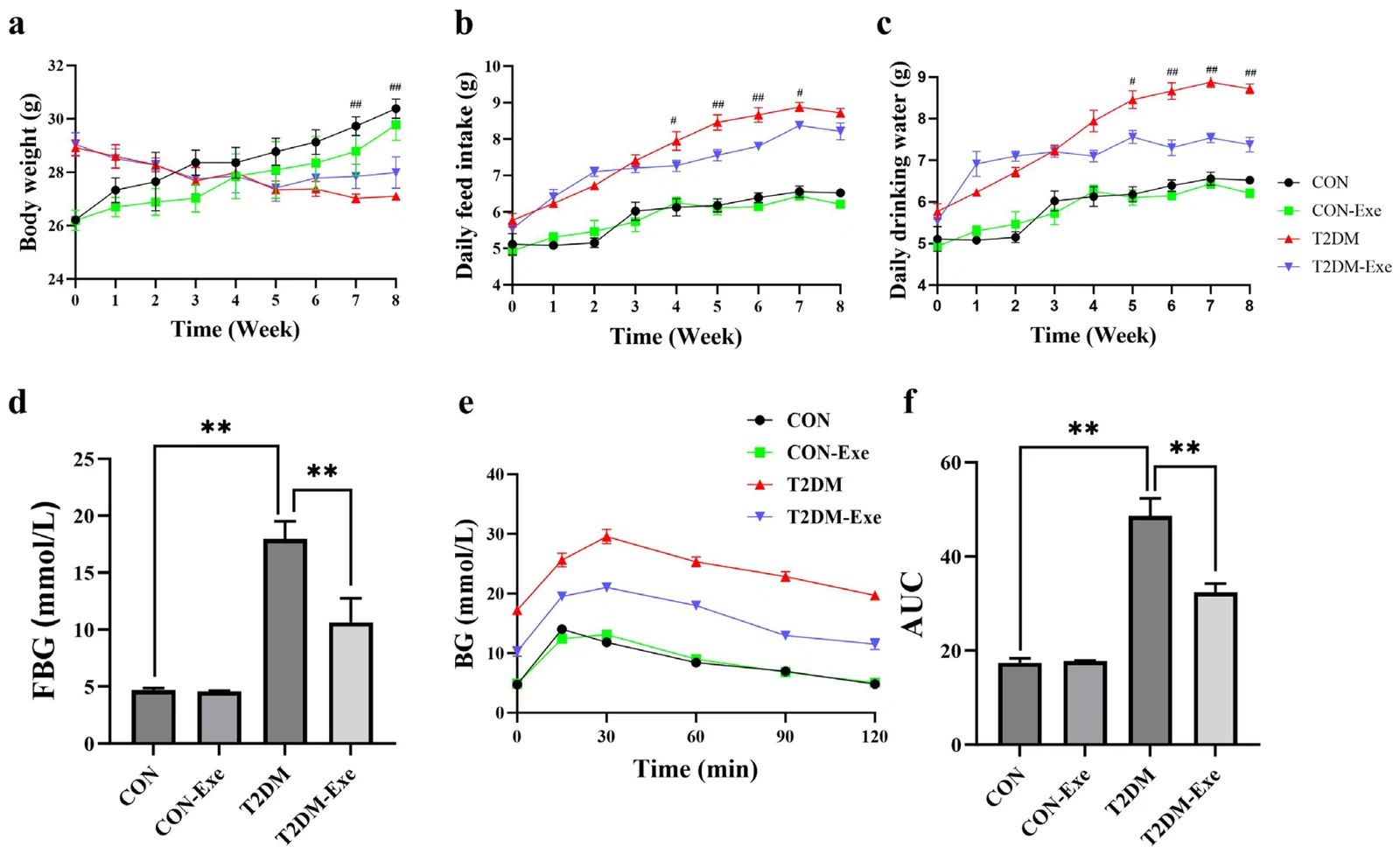
Effects of aerobic exercise on body weight, food/water intake, and glycemic parameters in T2DM mice. (**a**) Weekly changes in body weight; (**b**) weekly food intake; (**c**) weekly water consumption; (**d**) fasting blood glucose levels at the end of the intervention; (**e**) oral glucose tolerance test (OGTT); (**f**) area under the curve (AUC) calculated from the OGTT. Data are presented as mean ± SEM. In Figures (**a**–**c**), # *p* < 0.05, ## *p* < 0.01 indicate significant differences between T2DM and T2DM-Exe. In Figures (**d**–**f**), ** *p* < 0.01 indicates significant differences between T2DM and CON.

Our results demonstrated that, unlike the continuous weight gain observed in the CON group, T2DM mice exhibited a progressive decrease in body weight over the experimental period. By week 7, body weight in the T2DM-Exe group was significantly higher than that in the T2DM group (Figure 1a). Regarding food intake, T2DM-Exe mice consumed significantly less food than T2DM mice during weeks 4~7 of the aerobic exercise intervention (Figure 1b). For water consumption, T2DM mice showed a marked increase in drinking volume at week 5 compared with T2DM-Exe mice (*p* < 0.05; Figure 1c), whereas no statistically significant differences were observed between the CON-Exe and CON groups in any of the above parameters (body weight, water intake, or food intake). Furthermore, fasting blood glucose levels and oral glucose tolerance test (OGTT) results were significantly elevated in T2DM mice relative to CON mice (*p* < 0.01; Figure 1d–f). In contrast, both fasting blood glucose and the area under the curve (AUC) were significantly improved in T2DM-Exe mice compared with T2DM mice (*p* < 0.01; Figure 1d–f). Collectively, these findings indicate that aerobic exercise effectively ameliorates glucose metabolism in type 2 diabetes and alleviates typical symptoms such as polydipsia and polyphagia.

### 2.2. Exercise Improves Aortic Morphology in T2DM Mice

**Figure 2:**
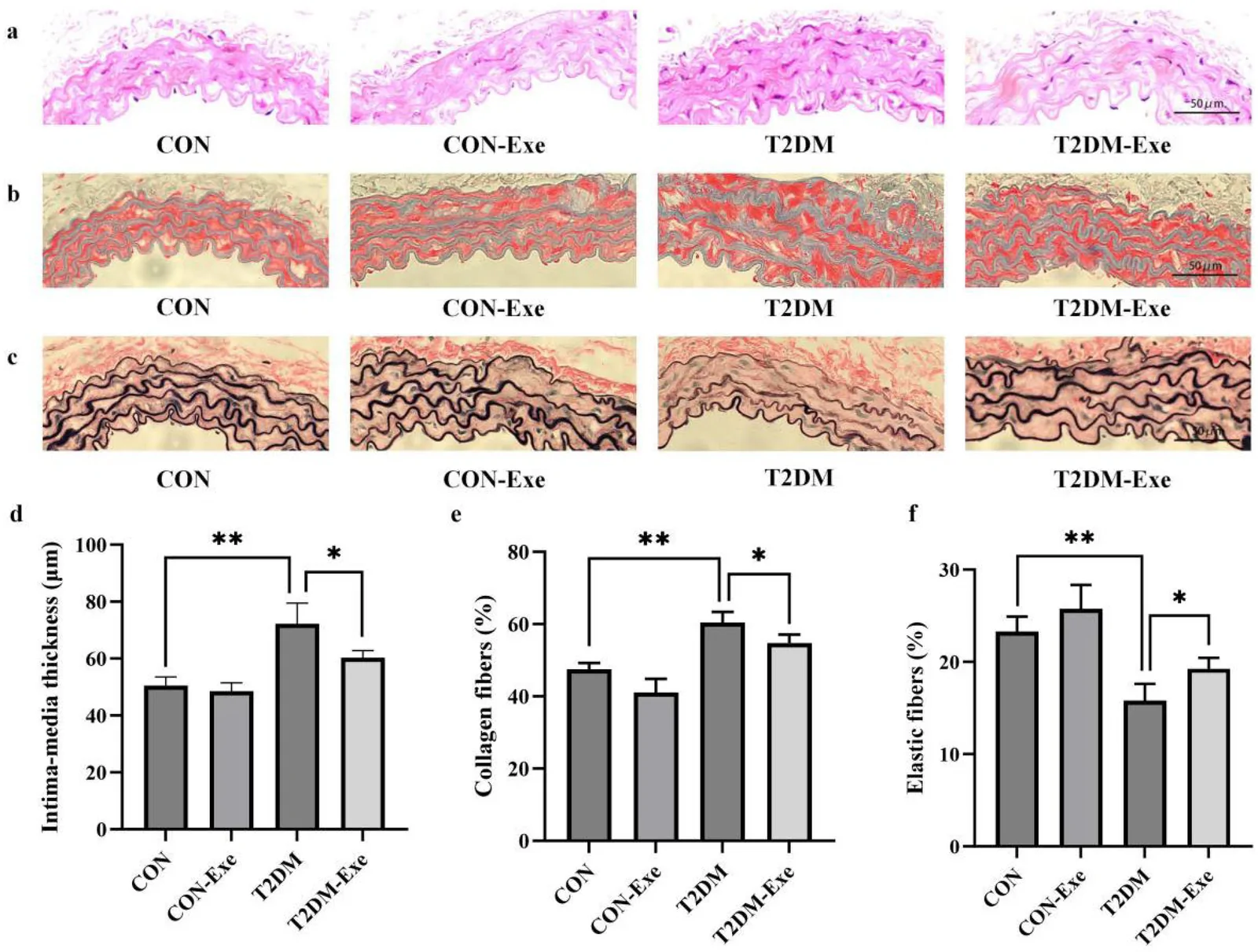
Aerobic exercise significantly ameliorated arterial wall thickening, excessive collagen deposition, and elastic fiber fragmentation in the thoracic aorta of T2DM mice. Scale bar: 50 μm. (**a**) Representative images of Hematoxylin–Eosin (HE) staining; (**b**) representative images of Masson staining; (**c**) representative images of Verhoeff-Van Gieson (VVG) staining, each performed on cross-sections of the thoracic aortic wall. Quantitative analyses revealed that aerobic exercise markedly reduced (**d**) medial thickness, (**e**) collagen fiber area fraction, and (**f**) elastic fiber degradation in the thoracic aortic wall of T2DM mice. Data are presented as mean ± SEM; * *p* < 0.05, ** *p* < 0.01.

The most prominent feature of vascular remodeling is thickening of the vascular wall. HE staining revealed that the thoracic aorta wall in the T2DM group was significantly thickened compared with that in the CON group (*p* < 0.01; Figure 2a,d). After 8 weeks of aerobic exercise intervention, the vascular wall thickness in the T2DM-Exe group was significantly reduced relative to the T2DM group (*p* < 0.05), whereas no significant difference was observed between the CON and CON-Exe groups. Masson staining showed a marked increase in collagen fibers in the arterial wall of T2DM mice versus CON mice (*p* < 0.01; Figure 2b,e). In contrast, the T2DM-Exe group exhibited a significant decrease in collagen fibers compared with the T2DM group (*p* < 0.05), indicating that aerobic exercise effectively mitigates collagen deposition in the vascular wall. VVG staining results demonstrated that arterial elastic fibers were significantly decreased in the T2DM group compared with the CON group (*p* < 0.01; Figure 2c,f). Aerobic exercise significantly augmented elastic fibers in the T2DM-Exe group versus the T2DM group (*p* < 0.05). Collectively, these findings suggest that aerobic exercise inhibits pathological arterial wall thickening and preserves extracellular matrix homeostasis in T2DM mice.

### 2.3. Exercise Alleviates Mitochondrial Damage and Regulates the Phenotype of VSMCs in T2DM Mice

**Figure 3:**
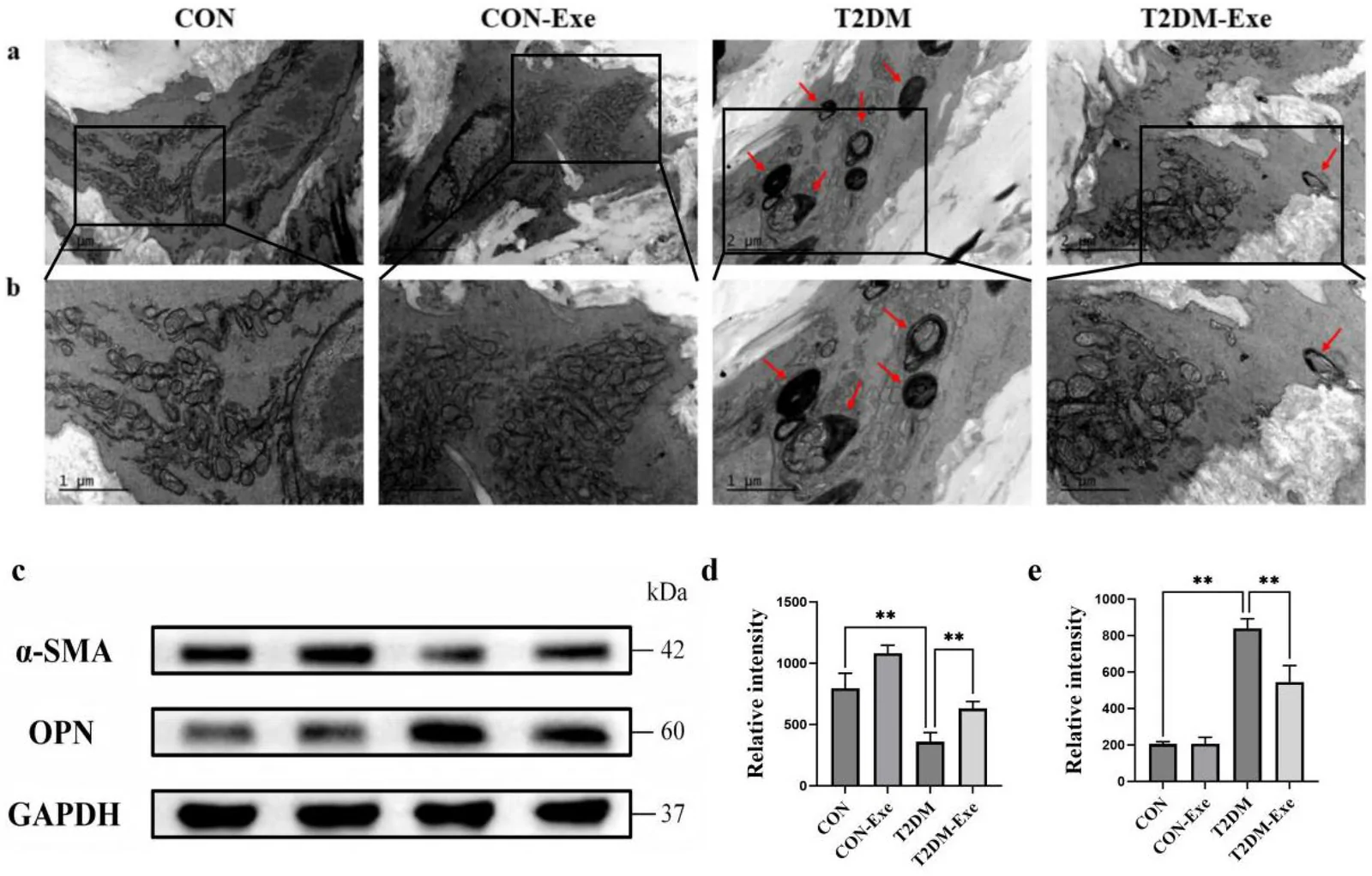
Exercise attenuated VSMC phenotypic switching and restored mitochondrial morphology in T2DM mice. (**a**,**b**) Mitochondrial ultrastructure in the medial layer (TEM); red arrows indicate mitophagosomes, and black boxes indicate regions shown at higher magnification. Scale bars: 2 μm in (**a**) and 1 μm in (**b**). (**c**) Representative blots for α-SMA and OPN, with densitometric quantification (full blots in Supplementary Materials). (**d**,**e**) Quantification of α-SMA and OPN expression, respectively. Data are presented as mean ± SEM. ** *p* < 0.01.

Mitochondria are core organelles that maintain cellular energy metabolism and cell survival. Transmission electron microscopy was used to assess the morphological and pathological alterations of mitochondria in mouse thoracic aortic VSMCs across the different experimental groups. Compared with the CON group, the T2DM group exhibited markedly aggravated mitochondrial damage in VSMCs, characterized by obvious swelling, vacuolization, and severe disruption of mitochondrial cristae, with some cristae even disappearing completely (Figure 3a,b). However, following an 8-week aerobic exercise intervention, the mitochondrial damage in the T2DM-Exe group was significantly attenuated, with mitochondrial morphology largely restored to normal and cristae structures clearly discernible. Collectively, these findings suggest that aerobic exercise alleviates mitochondrial morphological damage in VSMCs of T2DM mice. As shown in Figure 3c–e, the protein expression level of α-SMA was significantly lower in the T2DM group than in the CON group (*p* < 0.01), whereas the OPN protein level was significantly higher (*p* < 0.01). After exercise intervention, the α-SMA protein level in the T2DM-Exe group was significantly increased compared with that in the T2DM group (*p* < 0.01), whereas the OPN protein level was significantly decreased (*p* < 0.01). These results indicate that aerobic exercise effectively inhibits the phenotypic switching of aortic smooth muscle cells in T2DM mice. No significant differences were observed in the protein expression levels of α-SMA or OPN in VSMCs between the CON-Exe and CON groups.

### 2.4. Exercise Regulates the Expression of Sirt1 in T2DM Mice

**Figure 4:**
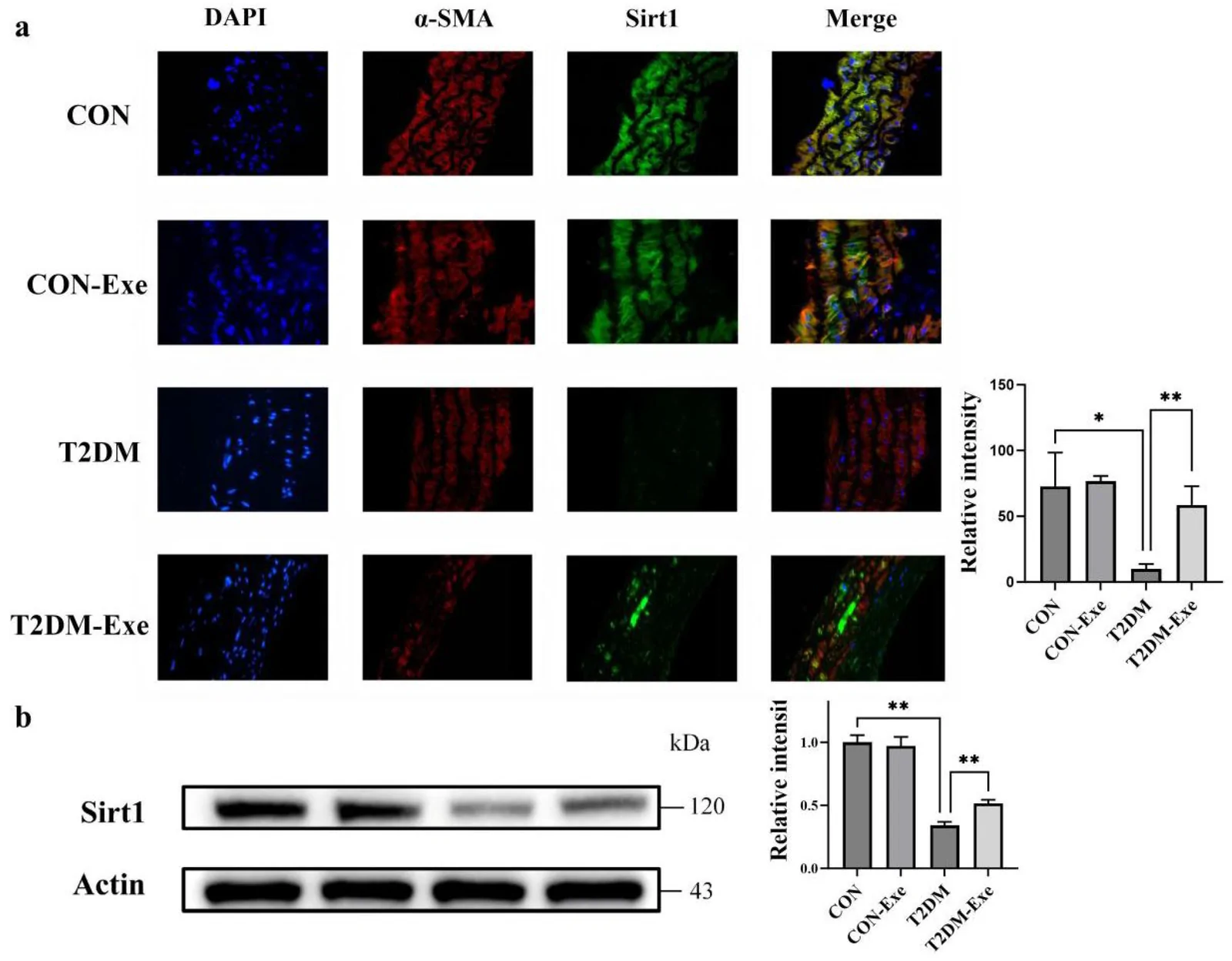
Immunofluorescence staining of Sirt1 (green) in the aortic smooth muscle layer of mice from the CON, T2DM, T2DM-Exe, and CON-Exe groups after 8-week intervention. (**a**) Representative images of Sirt1 expression and Quantification of Sirt1 fluorescence intensity; (**b**) representative blots for Sirt1, with densitometric quantification. Data are shown as mean ± SEM. * *p* < 0.05, ** *p* < 0.01.

The protein level of Sirt1 was significantly reduced in the T2DM group compared with the CON group (Figure 4a,b), whereas no significant difference was detected between the CON-Exe and CON groups. In contrast, the Sirt1 protein level in the T2DM-Exe group was significantly increased compared with that in the T2DM group (Figure 4a,b). These findings indicate that aerobic exercise restores Sirt1 expression in VSMCs of T2DM mice.

### 2.5. Effect of Exercise on Glucose Metabolism of VSMCs in T2DM Mice

**Figure 5:**
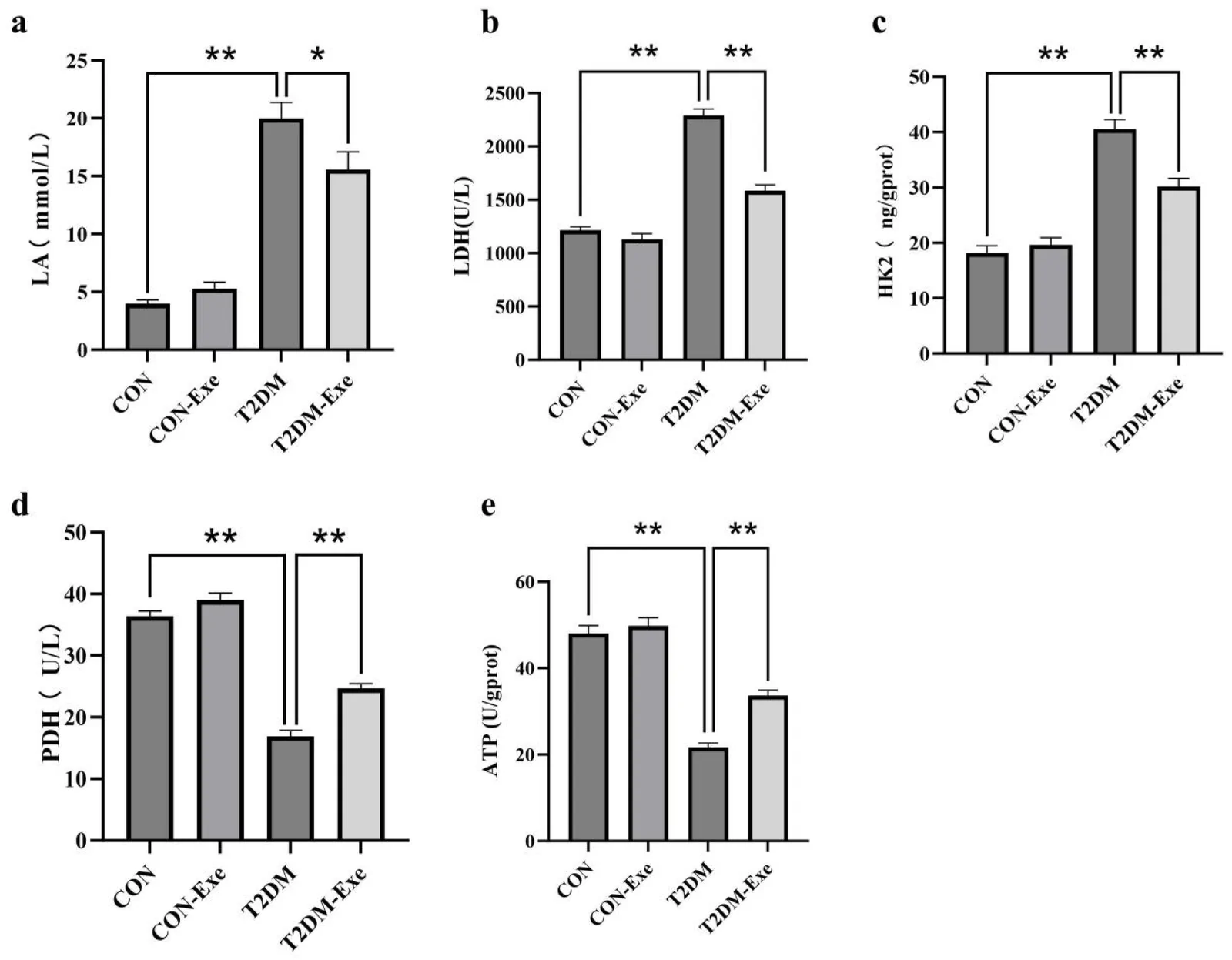
Effects of aerobic exercise on glucose metabolism in the aortic smooth muscle of T2DM mice. Levels of (**a**) L-lactate (LA) and ATP (**e**), together with the activities of (**b**) lactate dehydrogenase (LDH), (**c**) hexokinase 2 (HK2), and (**d**) pyruvate dehydrogenase (PDH), were determined in aortic smooth muscle tissue. Data are presented as mean ± SEM. * *p* < 0.05; ** *p* < 0.01.

Analysis of glucose metabolic enzymes showed that, compared with the CON group, the T2DM group exhibited significantly increased LDH activity (*p* < 0.01; Figure 5b), HK2 activity (*p* < 0.01; Figure 5c), and lactate levels (*p* < 0.01; Figure 5a) in the aortic smooth muscle layer. Conversely, pyruvate dehydrogenase (PDH) activity and adenosine triphosphate (ATP) levels, indicators of oxidative metabolism, were significantly decreased (both *p* < 0.01; Figure 5d,e). Following aerobic exercise intervention, the T2DM-Exe group exhibited significantly lower HK2 activity (*p* < 0.01), LDH activity (*p* < 0.01), and lactate levels (*p* < 0.05), together with significantly higher PDH activity (*p* < 0.01) and ATP levels (*p*< 0.01), compared with the T2DM group. These results were accompanied by increased PDH activity and ATP levels and decreased HK2 activity, LDH activity, and lactate accumulation following aerobic exercise. No significant differences in glucose metabolic enzyme activities or metabolite levels were observed between the CON and CON-Exe group.

## 3. Discussion

The present study systematically investigated the effects of 8-week treadmill aerobic exercise on vascular remodeling in T2DM mice, with a particular focus on the phenotypic modulation of aortic VSMCs and its underlying metabolic mechanisms. Our findings demonstrate that aerobic exercise significantly improved glucose metabolism and ameliorated polydipsia and polyphagia in T2DM mice, attenuated vascular wall thickening, collagen deposition, and elastic fiber degradation, inhibited the phenotypic switching of VSMCs from contractile to synthetic phenotype, restored mitochondrial ultrastructure, upregulated Sirt1 expression in the aortic smooth muscle layer, and rebalanced glycolytic and oxidative phosphorylation metabolic fluxes. Collectively, these results provide comprehensive experimental evidence that aerobic exercise exerts multi-target protective effects against diabetic vascular remodeling, potentially through the Sirt1-mediated regulation of VSMC phenotype and glucose metabolic reprogramming.

Consistent with previous reports, our study confirmed that T2DM mice exhibited typical diabetic symptoms including hyperglycemia, polydipsia and polyphagia. Notably, 8 weeks of aerobic exercise intervention significantly alleviated these metabolic disturbances, as evidenced by improved blood glucose control and reduced food and water intake in the T2DM-Exe group compared with the T2DM group. These findings align with the well-established role of regular aerobic exercise in enhancing insulin sensitivity and promoting glucose uptake and utilization in peripheral tissues [9]. The beneficial metabolic effects of exercise are largely attributed to the activation of Adenosine 5‘-monophosphate (AMP)-activated protein kinase (AMPK)/SIRT1/ Peroxisome proliferator-activated receptor γ coactivator 1α (PGC-1α) signaling axis, which serves as a central regulator of skeletal muscle metabolic homeostasis and mitochondrial biogenesis. Hyperglycemia and insulin resistance are major initiating factors in diabetes-associated vascular injury. Persistent exposure to high glucose concentrations can promote vascular dysfunction through several interconnected mechanisms, including activation of the polyol and hexosamine pathways, formation of advanced glycation end products (AGEs), activation of protein kinase C, oxidative stress, and chronic inflammation [10]. In addition to impairing endothelial function, the diabetic metabolic environment directly affects VSMCs contraction, proliferation, migration, and extracellular matrix production [11]. These changes collectively contribute to arterial wall thickening, fibrosis, and reduced vascular compliance. Although the primary focus of this study is vascular remodeling, the systemic metabolic improvements induced by exercise likely create a less hostile biochemical environment for the vascular wall, thereby reducing the sustained hyperglycemic stimulus that drives VSMC dysfunction.

Diabetic vasculopathy is characterized by structural alterations of the vascular wall, including increased intima-media thickness (IMT), excessive collagen deposition, and degradation of elastic fibers. In the present study, histological analyses (HE, Masson and VVG staining) revealed that T2DM mice exhibited significant vascular wall thickening, disorganized and fragmented elastic fibers, and markedly increased collagen deposition. These pathological changes are consistent with the recognized features of diabetic vascular remodeling, in which chronic hyperglycemia directly stimulates VSMC proliferation and migration, leading to vascular wall thickening, while excessive collagen accumulation promotes vascular fibrosis and stiffening.

Elastin, which constitutes the structural backbone of elastic fibers, is normally deposited and cross-linked within the medial layer of the vascular wall under physiological conditions. Beyond contributing to vascular structural integrity and elasticity, elastin exerts an inhibitory effect on VSMC proliferation and migration, thereby maintaining the contractile phenotype of VSMCs. In the diabetic state, degradation or fragmentation of elastic fibers compromises this regulatory function and increases vascular stiffness. Conversely, collagen, a protein characterized by its tensile strength and rigidity, plays a critical role in limiting vascular distensibility. Abnormal collagen deposition is a hallmark of pathological vascular remodeling and contributes to reduced vascular compliance and hemodynamic alterations [12].

Our finding that aerobic exercise simultaneously reduced aortic wall thickening and collagen deposition while preserving elastic fibers suggests that its beneficial effects extend beyond a single component of the vascular wall. Previous experimental and clinical studies have shown that regular exercise can improve arterial stiffness, endothelium-dependent vasodilation, and vascular inflammation. The present findings provide additional histological evidence that aerobic exercise protects against diabetes-related aortic remodeling. Several mechanisms may account for these effects. First, exercise-induced improvements in glucose metabolism may reduce AGE formation and collagen cross-linking. Second, exercise may attenuate oxidative stress and inflammation, thereby inhibiting profibrotic pathways such as transforming growth factor-β signaling. Third, suppression of VSMC proliferation, migration, and synthetic phenotypic switching may reduce excessive extracellular matrix production. Finally, improved endothelial production of nitric oxide and other vasoprotective mediators may reduce pathological stimulation of medial VSMCs.

The phenotypic transition of VSMCs from a contractile to a synthetic phenotype is a key pathological event in diabetic vascular remodeling, contributing to arterial stiffness, neointima formation, and plaque development. α-SMA, a cytoskeletal protein essential for vasoconstriction, serves as a specific marker of the contractile phenotype, whereas OPN, a highly phosphorylated secretory glycoprotein, is widely expressed in the extracellular matrix and is strongly upregulated under pathological conditions, marking the synthetic phenotype.

In this study, Western blot analysis revealed that T2DM mice exhibited significantly decreased α-SMA expression and increased OPN expression in aortic VSMCs, indicative of a pathological shift toward the synthetic phenotype. Notably, aerobic exercise intervention effectively reversed this trend, as evidenced by upregulated α-SMA and downregulated OPN in the T2DM-Exe group, suggesting that exercise partially restored the differentiated contractile phenotype and suppressed the synthetic, proliferative, and matrix-producing properties of VSMCs.

Mitochondria are central to biosynthesis, bioenergetics and biological signal transduction, and their morphological integrity is essential for normal cellular function. Alterations in mitochondrial morphology can lead to metabolic dysfunction and mitochondrial dynamic disorders, which in turn promote excessive proliferation and migration of VSMCs. Transmission electron microscopy demonstrated abnormal mitochondrial morphology in the aortic VSMCs of T2DM mice, whereas aerobic exercise preserved mitochondrial ultrastructure. Chronic hyperglycemia and oxidative stress can induce mitochondrial swelling, cristae disruption, loss of membrane integrity, and dysregulation of mitochondrial quality control. These abnormalities impair ATP production and increase mitochondrial reactive oxygen species generation, thereby activating inflammatory, apoptotic, and phenotypic switching pathways.

In VSMCs, mitochondrial dysfunction may establish a self-reinforcing cycle involving metabolic imbalance, oxidative stress, and phenotypic modulation. When mitochondrial oxidative phosphorylation is impaired, cells may increase glycolysis to maintain energy production. However, increased glycolytic activity, lactate accumulation, reactive oxygen species production, and altered metabolite availability can influence transcription factor activity, chromatin modifications, and extracellular matrix synthesis. These changes may favor VSMC proliferation and the acquisition of a synthetic phenotype [13]. Conversely, the increased biosynthetic and energetic demands of synthetic VSMCs may further promote metabolic reprogramming.

Aerobic exercise can enhance mitochondrial biogenesis, improve the balance between mitochondrial fusion and fission, facilitate the removal of damaged mitochondria, and strengthen antioxidant defenses. The Sirt1–PGC-1α axis is a plausible pathway through which exercise regulates mitochondrial function. Exercise-induced metabolic stress can activate AMPK and alter the intracellular NAD^+^/NADH ratio, thereby promoting Sirt1 activity. Sirt1 can subsequently deacetylate PGC-1α and other substrates involved in mitochondrial biogenesis and oxidative metabolism. Thus, the exercise-induced improvement in mitochondrial morphology observed in this study may be functionally related to increased Sirt1 expression and the restoration of oxidative metabolism.

Sirt1, a class III histone deacetylase, has emerged as a key regulator of vascular homeostasis and a critical mediator of the beneficial effects of exercise [14]. Experimental evidence across endothelial cells, macrophages and VSMCs consistently demonstrates that SIRT1 activation preserves nitric oxide bioavailability, suppresses ROS-dependent inflammasome signaling, modulates macrophage polarization, inhibits ferroptosis and maintains mitochondrial integrity [15,16]. Importantly, SIRT1 has been shown to inhibit VSMC proliferation and migration, induce cell cycle arrest at the G1/S transition, and suppress neointima formation following vascular injury [17].

In this study, immunofluorescence staining and Western blot analysis demonstrated that Sirt1 protein expression in the aortic smooth muscle layer was significantly increased in the T2DM-Exe group compared with the T2DM group. This finding suggests that aerobic exercise is associated with the upregulation of Sirt1 expression in the diabetic vascular wall, accompanied by reduced vascular remodeling and attenuation of VSMC phenotypic switching. Collectively, these results support an association between exercise-induced Sirt1 upregulation and an improved vascular smooth muscle phenotypic profile, although the present data do not establish a direct causal relationship.

The alteration of glucose metabolism is a critical driver of VSMC phenotypic switching. Under pathological conditions, VSMCs undergo a metabolic shift reminiscent of the “Warburg effect,” characterized by enhanced glycolytic flux that supports proliferation, migration and phenotypic modulation. Increased glycolytic flux inhibits the expression of pro-apoptotic proteins, thereby promoting VSMC proliferation and migration [18,19].

T2DM mice exhibited increased HK2 and LDH activities and elevated lactate concentrations in aortic tissue. These changes were accompanied by abnormalities in PDH-related metabolism and ATP production. Aerobic exercise reduced glycolysis-related enzyme activity and lactate accumulation while improving PDH-related metabolism and cellular energy status. These findings indicate that the diabetic aorta develops a metabolic profile characterized by increased glycolysis-related metabolism and impaired mitochondrial glucose oxidation, whereas exercise partially corrects this metabolic imbalance.

HK2 catalyzes the first committed step in intracellular glucose metabolism and is an important determinant of glycolytic activity. LDH catalyzes the reversible conversion of pyruvate to lactate and contributes to the maintenance of cytosolic redox balance during increased glycolysis. Elevated lactate is not merely a passive end product of glycolysis but may also influence cellular behavior by altering redox balance, signaling pathways, and epigenetic modifications, including histone lactylation. Such changes can affect inflammation, proliferation, and extracellular matrix production. In VSMCs, enhanced glycolysis may provide both rapidly available ATP and biosynthetic intermediates required for proliferation, migration, and collagen synthesis. Glycolytic reprogramming may therefore facilitate the acquisition and maintenance of the synthetic phenotype.

PDH is a critical metabolic checkpoint linking cytosolic glycolysis to the mitochondrial tricarboxylic acid cycle. Reduced PDH activity limits the entry of pyruvate into mitochondrial oxidative metabolism and favors its conversion to lactate. Thus, the concurrent increases in HK2 and LDH activities and lactate levels, together with altered PDH-related metabolism and ATP homeostasis, are consistent with a shift away from mitochondrial glucose oxidation toward glycolysis-related metabolism in the diabetic aorta. This shift may represent an important mechanistic link between mitochondrial dysfunction and VSMC phenotypic switching.

Aerobic exercise generally increases mitochondrial oxidative capacity and metabolic efficiency, promotes pyruvate oxidation, and reduces excessive reliance on glycolysis. The exercise-induced reductions in HK2 and LDH activities and lactate concentrations, together with improved PDH-related metabolism and ATP homeostasis, suggest that exercise restores the balance between glycolysis and oxidative phosphorylation. This metabolic correction may reduce the energetic and biosynthetic support required for synthetic VSMC activation, thereby contributing to the maintenance of a contractile phenotype.

## 4. Materials and Methods

### 4.1. Animal Experiments

*Male C57BL/6J mice* were purchased from Sibeifu Laboratory Animal Technology Co., Ltd. (SYXK SU 2022-0012, Beijing, China), aged 6 weeks and weighing 17~20 g, and were randomly assigned to two groups: fed either a high-fat diet (HFD) or a maintenance diet (MD) for 4 weeks. HFD (D12492, Beijing Keao Xieli Feed Co., Ltd., Beijing, China) mice were intraperitoneally injected with streptozotocin (STZ, 50 mg/kg body weight; Sigma-Aldrich, St. Louis, MO, USA) for 4 consecutive days. After fasting for 12 h, the fasting blood glucose (FBG) of the mice was detected by Sinonuo Bio-glucose meter (Sinonuo Bio-Sensing Co., Ltd., Changsha, China). When FBG exceeded 11.1 mmol/L twice a week, the model was considered to be successful. Three days post-STZ injection, the mice were confirmed as diabetic. After successful establishment of the T2DM model, mice were randomly assigned to the CON, CON-Exe, T2DM, and T2DM-Exe groups using a random allocation method to minimize selection bias: (1) normal diet group (control group, CON). (2) CON Exercise group (CON-Exe); (3) HFD model (T2DM model group, T2DM); and (4) T2DM Exercise group (T2DM-Exe) intervention group. Mice performed treadmill exercise for 60 min/day, 5 days/week, for 8 weeks (14 m/min, 0° incline). All animals were housed under controlled conditions, with a temperature of 22 ± 1 °C, relative humidity of 50 ± 10%, and a 12-h light/dark cycle. They were given appropriate diets and had ad libitum access to tap water. Mice were deeply anesthetized via intraperitoneal injection of sodium pentobarbital (50 mg/kg body weight). After confirming the loss of consciousness and absence of response to toe pinch, animals were euthanized by cervical dislocation. Death was confirmed by the absence of spontaneous respiration and cardiac arrest. All efforts were made to minimize animal suffering throughout the procedures. The thoracic cavity was rapidly dissected under a dissecting microscope to fully expose the heart and aorta, and the aortic endothelium and peripheral adipose tissue were rapidly dissected in precooled PBS solution.

### 4.2. Histological and Immunofluorescence Analysis

Vascular samples were fixed in 4% paraformaldehyde and subsequently embedded in paraffin. The embedded tissues were sectioned to a thickness of 4 μm and stained using hematoxylin-eosin (H&E), Masson, and Verhoeff-Van Gieson (VVG) kits (Servicebio, Wuhan, China) for histological analysis. Aortic thickness was measured using Cellsens Standard software (V4.4.1). Image analysis software (Image-Pro Plus 6.0) was used for quantitative statistical analysis of Masson-stained sections of the mouse aorta. The integral optical density (IOD) value was used for optical density analysis. In the section images, collagen fibers are represented in blue and smooth muscle fibers in red. Quantitative statistical analysis of VVG-stained sections was also performed using Image-Pro Plus 6.0, with optical density analysis based on IOD values. In these images, elastic fibers appear black and smooth muscle fibers appear yellow. For Immunofluorescence analysis, the dewaxed sections were hydrated for antigen recovery and incubated with 0.3% Triton X-100. After the slices were incubated overnight with the primary antibody, the corresponding fluorescent secondary antibody was incubated. 4′,6-diamidino-2-phenylindole (DAPI) was used as a nuclear stain. Images were taken with a fluorescence microscope.

### 4.3. Transmission Electron Microscope

The vascular wall was initially fixed overnight in 0.1 M cacodylate buffer containing 2.5% glutaraldehyde and 100% Tween 20. After rinsing in the buffer, specimens were post-fixed for 2 h and dehydrated using a graded ethanol series. The specimens were then infiltrated sequentially with acetone, resin, and pure resin at 33 °C, followed by embedding in pure resin. Polymerization was carried out at 37 °C, 50 °C, and 60 °C for 12 h at each temperature. Ultrathin sections (60–80 nm) were cut with a glass knife, mounted on copper grids, and stained with uranyl acetate and lead citrate for 8 min. The sections were examined using a Hitachi HT7800 transmission electron microscope (Hitachi High-Tech, Tokyo, Japan) operated at 120 kV.

### 4.4. Western Blots

Western blot analysis of protein expression was conducted as described previously [7]. Briefly, the medial layer, denuded of the endothelium and adventitia, was lysed with RIPA buffer (Servicebio, Wuhan, China), and the protein concentration was determined using a BCA kit. The primary antibodies and their concentrations used for Western blotting were: Sirt1 (1:1000, NBP2-27205, Novus Biologicals, Centennial, CO, USA), α-SMA (1:1000, GB111364, Servicebio, Wuhan, China), OPN (1:2000, AF0227, Affinity Biosciences, Cincinnati, OH, USA). Secondary antibodies were diluted 1:10,000 and incubated for 1 h at room temperature. The samples were then washed with PBST. The samples were visualized using enhanced chemiluminescence reagent (KF8005, Affinity Biosciences, Cincinnati, OH, USA) on a Gel Imaging System (ChemiScope Touch 6100, Shanghai, China). The intensity of each band was quantified with ImageJ software (Image-Pro Plus 6.0).

### 4.5. Biochemical Assays for Glycolytic Enzymes and Metabolites

To evaluate glycolytic flux and mitochondrial oxidative metabolism in the vascular wall, the aortic medial layer, denuded of the endothelium and adventitia, was dissected and homogenized in ice-cold phosphate-buffered saline (PBS, pH 7.4) using a tissue grinder. The homogenates were centrifuged at 12,000× *g* for 15 min at 4 °C, and the supernatants were collected for subsequent biochemical measurements. The activities of HK2, LDH, and PDH, as well as the concentrations of L-lactate (LA) and ATP, were determined using commercially available colorimetric assay kits (Elabscience Biotechnology Co., Ltd., Wuhan, China) following the manufacturer’s protocols. All samples were assayed in duplicate, and the results were normalized to the total protein content of the corresponding supernatants, which was quantified using a BCA protein assay kit (Servicebio, Wuhan, China).

### 4.6. Statistics

Data are expressed as mean ± Standard Error of the Mean (SEM) from at least three independent biological replicates per group. The comparison between two groups was performed using an unpaired Student’s *t*-test with GraphPad Prism software (V10). One-way or two-way analysis of variance (ANOVA) was applied to assess differences among three or more groups (* p < 0.05, ** p < 0.01). When ANOVA revealed significant differences, Tukey’s post hoc test was performed for multiple comparisons.

## 5. Integrated Perspective and Future Directions

Taken together, our findings provide an integrated perspective that aerobic exercise is associated with improved diabetic vascular remodeling through coordinated regulation of multiple biological processes: (1) systemic metabolic improvement, which may reduce hyperglycemic and metabolic stress contributing to vascular injury; (2) increased Sirt1 expression in the aortic smooth muscle layer, which was accompanied by improvements in VSMC phenotypic characteristics and vascular remodeling; (3) preservation of mitochondrial ultrastructural integrity and improved cellular bioenergetic status; and (4) restoration of glucose metabolic balance, characterized by reduced glycolysis-related alterations and enhanced oxidative metabolism. These combined adaptations were associated with attenuation of vascular wall thickening, reduced collagen deposition, preservation of elastic fibers, and maintenance of a more contractile VSMC phenotype.

While our study provides comprehensive evidence regarding the beneficial effects of aerobic exercise on diabetic vascular remodeling, several limitations should be acknowledged. First, although exercise-induced Sirt1 upregulation was observed and accompanied by improvements in vascular remodeling, mitochondrial characteristics, and metabolic alterations, the present study did not include Sirt1-specific loss-of-function or gain-of-function approaches. Therefore, a direct causal role of Sirt1 in mediating the vascular protective effects of exercise cannot be conclusively established. Future studies using cell-specific Sirt1 manipulation models are needed to further clarify its functional contribution. Second, the involvement of other sirtuin family members or additional molecular pathways in exercise-induced vascular adaptation remains to be investigated. Third, the translational relevance of these findings to human diabetic vascular complications requires further validation in clinical studies. Nevertheless, our findings provide experimental evidence supporting aerobic exercise as a beneficial non-pharmacological strategy for improving diabetic vascular remodeling.

## 6. Conclusions

In summary, this study demonstrates that 8 weeks of treadmill aerobic exercise effectively ameliorates diabetic vascular remodeling in T2DM mice, accompanied by restored aortic Sirt1 expression, attenuation of pathological VSMC phenotypic switching from the contractile to synthetic phenotype, and improved glucose metabolic balance characterized by reduced glycolytic flux and enhanced oxidative metabolism. These beneficial effects are further associated with preserved mitochondrial ultrastructure, reduced vascular wall thickening, decreased collagen deposition, and improved elastic fiber integrity.

## Data Availability

The datasets supporting the conclusions of this article are included within the article and its Supplementary File.

## Supplementary Materials

The following supporting information can be downloaded at: https://www.mdpi.com/article/10.3390/ijms27198750/s1.
